# Spatial Variability and Distribution of the Metals in Surface Runoff in a Nonferrous Metal Mine

**DOI:** 10.1155/2016/4515673

**Published:** 2016-03-16

**Authors:** Bozhi Ren, Yangbo Chen, Guocheng Zhu, Zhenghua Wang, Xie Zheng

**Affiliations:** College of Civil Engineering, Hunan University of Science & Technology, Xiangtan 411201, China

## Abstract

The spatial variation and distribution features of the metals tested in the surface runoff in Xikuangshan Bao Daxing miming area were analyzed by combining statistical methods with a geographic information system (GIS). The results showed that the maximum concentrations of those five kinds of the metals (Sb, Zn, Cu, Pb, and Cd) in the surface runoff of the antimony mining area were lower than the standard value except the concentration of metal Ni. Their concentrations were 497.1, 2.0, 1.8, 22.2, and 22.1 times larger than the standard value, respectively. This metal pollution was mainly concentrated in local areas, which were seriously polluted. The variation coefficient of Sb, Zn, Cu, Ni, Pb, and Cd was between 0.4 to 0.6, wherein the Sb's spatial variability coefficient is 50.56%, indicating a strong variability. Variation coefficients of the rest of metals were less than 50%, suggesting a moderate variability. The spatial structure analysis showed that the squared correlation coefficient (*R* ^2^) of the models fitting for Sb, Zn, Cu, Ni, Pb, and Cd was between 0.721 and 0.976; the ratio of the nugget value (*C*_0_) to the abutment value (*C* + *C*_0_) was between 0.0767 and 0.559; the semivariogram of Sb, Zn, Ni, and Pb was in agreement with a spherical model, while semivariogram of Cu and Cd was in agreement with Gaussian model, and both had a strong spatial correlation. The trend and spatial distribution indicated that those pollution distributions resulting from Ni, Pb, and Cd are similar, mainly concentrated in both ends of north and south in eastern part. The main reasons for the pollution were attributed to the residents living, transportation, and industrial activities; the Sb distribution was concentrated mainly in the central part, of which the pollution was assigned to the mining and the industrial activity; the pollution distributions of Zn and Cu were similar, mainly concentrated in both ends of north and south as well as in west; the sources of the metals were widely distributed.

## 1. Introduction

The release and migration of the metals (e.g., Sb, Ni, Cu, Zn, Cd, and Pb) often result from the nonferrous metal mining, smelting and transportation, waste slag dumps for long-term weathering, and rain leaching. The heavy metals usually flow into mining area via surface runoff, and subsequently to local streams, rivers, lakes, and underground water. This would cause a serious heavy metal pollution to ecological environment, thus posing a potential threat to human health. Furthermore, the threat is also posed towards the sustainable development of local economy, which is dependent on the ecological environment [1–3].

The mechanism and ecological risk assessment of soil heavy metal pollution resulting from the metal mines have received considerable attention, especially in the investigation, analysis, and assessment of water environment pollution in the mining area [4–8]. However, research on the characteristics of the surface runoff pollution to the regional water environment is rare, especially on the spatial variation and the distributions of heavy metals in surface runoff. The geostatistical methods have been successfully applied to the spatial variability analyses of sand gravel content as well as the pH value in soil [9]. With the development of geostatistical theory, it has been successfully applied to various fields of the environmental science and a significant achievement has also been obtained [10–14]. For example, the spatial structure characteristics of the water content of soil as well as conductivity, nitrogen and phosphorus, organic matter and polycyclic aromatic hydrocarbons, metals and other pollutants, and even spatial characteristics of the pollutants in the atmospheric environment were investigated using the geostatistical methodologies. Considering the mutual penetration among disciplines and the developments of statistical software and geographical information system (GIS), the statistical methods have been widely applied in the analyses of spatial variation and structural characteristics of the metal content of soil, which resulted from lake sediment, irrigation and reservoir upstream, typical mining cities and urban fringe chemical industry park, city suburbs, and urban areas [5, 15–23]. In addition, the significant applications in the determination of metal sources have been studied. However, few studies on the spatial variation and distribution characteristics of heavy metals on surface runoff in nonferrous metal mines were reported.

In China, the mining industries have developed rapidly, among which the antimony ore mining is the most developed industry because its production capacity is the highest. However, during mine exploitation, human activities, such as mining, smelting, and transport, have led to a serious environmental pollution in antimony mine area, especially the pollution to the water environment and the residents' living, thus influencing the development of local economy. In this study, Baodaxing mining area that is located in Hunan, China, was selected as research objective. The geostatistical methods were used for the investigation of the spatial variability and the distribution characteristics of the metals within mining area in combination with GIS. This research aimed to provide a technique support of treatment of metal pollution to the water environment and of comprehensive control for antimony mining area.

## 2. Materials and Methods

### 2.1. Monitoring Area and Sampling

**Figure 1:**
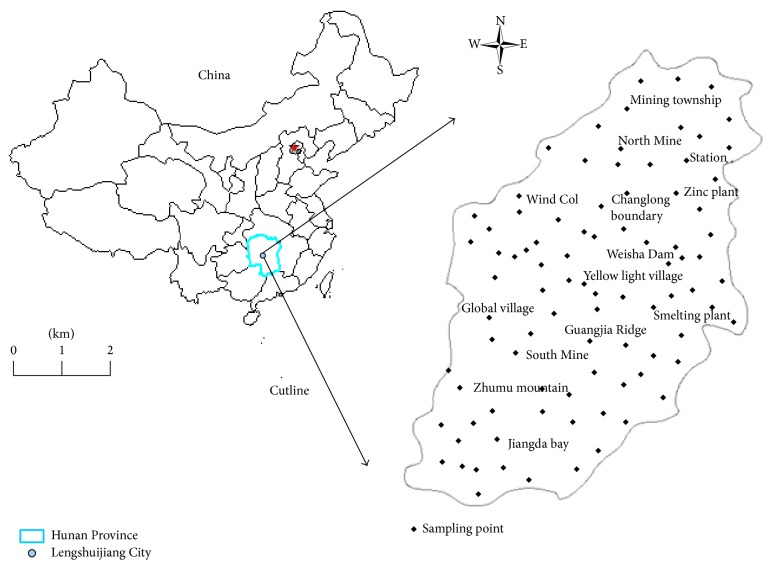
Sampling points distributions.

Baodaxing mining, is located in south of Tin Mine, Lengshuijiang City, Hunan, China, with geographical coordinates between 111°25′47′′ and 111°31′22′′ and longitude from 27°49′28′′ to 27°43′05′′. There are numerous low mountains and hills with altitude ranging from 300 to 600 m and with area up to 4.826 km^2^. The climate is moist with average temperature of 16.7°C. It is cool in spring and summer while the temperature within fall and winter is relatively lower. The maximum wind speed can be up to 17 m/s. The rainy season often occurs in summer. In recent 60 years during 1949 and 2008, the average rainfall capacity is up to 1381.6 mm. In this study, the sampling points were distributed with an interval of 0.03–0.06 km^2^ and there were total 94 sampling points. The distributions of sampling points are shown in Figure 1. WGS-84 and RTK instruments were used to record coordinate and elevation for each sampling point, respectively. The COORD 4.2 software was used to convert the parameters into a rectangular plane coordinate system. The experimental investigation was carried out throughout the whole year. Once the rainfall runoff occurred, the samples were collected from surface runoff. At each sampling point, 0.6–3 L water sample was collected in the polyethylene bottle, which was packed with black plastic bags and finally brought back to the lab for analysis.

### 2.2. Sampling

The water samples were shaken until homogeneous water samples were obtained, followed by a natural settlement of 20–30 mins. The upper nonsettlement was siphoned and filtered by 0.45 *μ*m filter membrane. The filtrate was acidified to pH < 2 via hydrochloric acid and nitric acid [1, 24]. According to the Chinese Standard Method for Drinking Water Testing (GB5750.6-2006), the atomic absorption spectroscopy (AA7003A, Beijing Dongxi Institute of Electronic Technology) was used to measure the Cu, Zn, Sb, Ni, Pb, and Cd. For low concentration water samples, they were concentrated prior to the test. The accuracy of the method for the measurements of the metals was calibrated via standard recovery experiment.

### 2.3. Geostatistical Analysis

The geostatistical analysis established based on the regional variables and semivariation function is often used to investigate the distribution variation and the characteristics of pollutants in regional space [15]. The pollutant variation is defined as a variable as *Z*(*x*); *x* is the position in which pollutant is located. The present work was to adopt the variation function to analyze the spatial variation and the distribution characteristics of metals in surface runoff. The variation function can be expressed as follows [25]:

(1) $$\begin{array}{c} \gamma \left(\;h\;\right)=\frac{1}{2N\left(h\right)}\overset{N\left(h\right)}{\underset{i=1}{\sum }}{\left[\;Z\left(\;x_{i}\;\right)-Z\left(\;x_{i}+h\;\right)\;\right]}^{2}, \end{array}$$

where *γ*(*h*) is the variation function; *h* is the step length (m); *N*(*h*) is the number of those sampling points where the distance between samples is equal to *h*; *Z*(*x*_*i*_) and *Z*(*x*_*i*_ + *h*) are measured pollutant values (mg/L) at *x*_*i*_ and *x*_*i*_ + *h*, respectively.

In geostatistical analysis, semivariation function contains the models without/with partial sill values. The Gauss model, spherical model, and exponential model belong to those with partial sill value. The most used model is spherical model, followed by Gauss model and exponential model. The Gauss model, spherical model, and exponential model are expressed using the following forms:

Spherical model:

(2) $$\begin{array}{c} \gamma \left(\;h\;\right)=\left\{\begin{array}{cc} 0, & h=0,\\ C_{0}+C\left(\frac{3h}{2a}-\frac{h^{3}}{2a^{3}}\right), & 0<h\leq a,\\ C_{0}+C, & h>a. \end{array}\right. \end{array}$$

Gauss model:

(3) $$\begin{array}{c} \gamma \left(\;h\;\right)=\left\{\begin{array}{cc} 0, & h=0,\\ C_{0}+C\left(1-e^{-h^{2}/a^{2}}\right), & h>0. \end{array}\right. \end{array}$$

Exponential Model:

(4) $$\begin{array}{c} \gamma \left(\;h\;\right)=\left\{\begin{array}{cc} 0, & h=0,\\ C_{0}+C\left(1-e^{-h/a}\right), & h>0, \end{array}\right. \end{array}$$

where *a* is a range march of which value corresponds to *x*-axis, standing for spatial self-correlation in investigated range. A larger *a* value implies a larger spatial self-correlation range; *C*_0_ is nugget value (semivariance given distance is zero); *C* is partial sill, standing for the variation from natural factors of landform, climate, and soil; *C*_0_ + *C* is partial sill value (it is *γ*(*h*) value under condition that the half-square difference tends to be stable, corresponding to the variable *a*), presenting the total variation of this variable in the variable range; in general, *C*_0_/(*C*_0_ + *C*) can be used to describe spatial correlation degree of specific variable in variable range. The ratio less than 25% means that the variable has a very strong spatial correlation, while that between 25% and 75% illustrates that it may have a moderate spatial correlation; however, beyond 75% it implies relatively weak spatial correlation [26].

### 2.4. Data Analysis

In this study, a scientific software (SPSS 19.0) was used in basic statistical analysis, normal distribution, and frequency analysis of the measured data. The Grubbs test method was used to remove the abnormal data from group. The normal distribution of the measured data was analyzed by Kolmogorov-Smirnov method [25, 27–30]. According to (1), the variation function was calculated using GS+V9.0 software. With the effective lag distance of 2 km and an acceptability angle of 22.5°, the fitting of the semivariance functions at different orientations (0°, 45°, 90°, and 135°) via Suffer 8.0 was carried out, which was used to analyze spatial variation of Ni, Cu, Sb, Cd, Zn, and Pb in surface runoff in the mining area. With the Kriging interpolation method in the ArcGIS 10.2, the spatial distribution of the above metals can be effectively analyzed [28].

## 3. Results and Discussion

### 3.1. Standard Recovery Experiment

The standard recovery experimental results are shown in Table 1. From Table 1, the recovery rate of Sb, Ni, Cu, Zn, Cd, and Pb were between 97.8% and 101.2%, which indicated that the measurement method of the metal samples utilized in this study was accurate and reliable.

### 3.2. Basic Statistical Analysis of Heavy Metals in Surface Runoff

**Figure 2:**
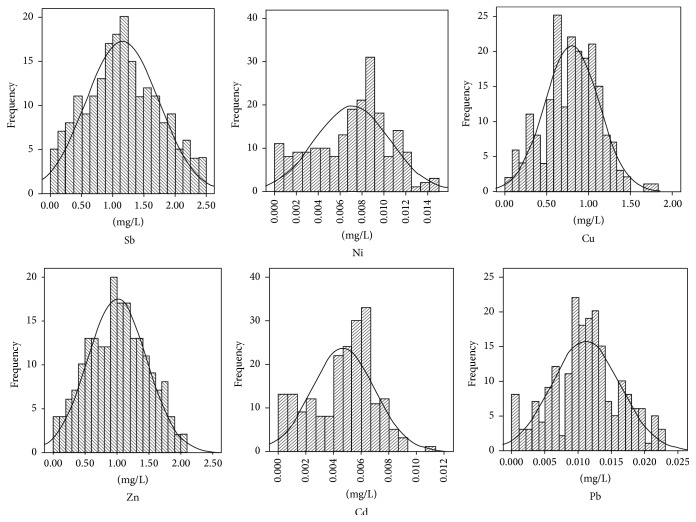
Frequency distributions of Sb, Ni, Cu, Zn, Cd, and Pb in surface runoff in antimony mine.

A comparison between the detection results of 94 samples from the investigated area and the metal index shown in the drinking water standard testing method (GB5750.6-2006) was made via SPSS software analysis. The results are shown in Table 2 and Figure 2.

It showed that the contents of Sb, Zn, Cu, Ni, Pb, and Cd in the surface runoff in the study area were in the range of 0.1315 to 2.4853 mg/L, 0.9161 to 2.0209 mg/L, 0.0403 to 1.7534 mg/L, 0.0001 to 0.0143 mg/L, 0.0001 to 0.2220 mg/L, and 0.0001 to 0.1105 mg/L, respectively. The difference between the maximum and the minimum of each element was relatively high. This showed that the six kinds of metals had an accumulation. As seen from the maximum and the standard values, except the maximum value of Ni lower than standard value, the maximum values of other five metals (Sb, Zn, Cu, Pb, and Cd) were far beyond standard value, which were 497.1, 2, 1.8, 22.2, and 22.1 times than the standard value, respectively, which showed that the five kinds of metals were mainly concentrated in the local region and their pollution was very serious. The excessive standard rate reflects the degree of excessive standard value. As seen from the excessive standard rate, it can be found that the excessive standard rates of Sb, Zn, Pb, and Cd were 100%, 50.51%, 60.29%, and 53.43%. All Sb samples exceeded the standard values, indicating that they have posed a serious pollution to the investigated area. Zn and Pb had about half of samples exceeding the standard values, of which pollution produced herein was only smaller than those resulting from Sb. Although about half of Cd samples exceeded the standard values, the Cd distribution within the investigated area was wider and thus the pollution was not significant. Ni having a smaller excessive standard rate of zero resulted in less pollution. The coefficients of the variation of the six kinds of metals ranged from 0.4 to 0.6. The spatial variation coefficient of Sb reached 50.56%, which belonged to strong variation type, suggesting that the spatial distribution of Sb in surface runoff was extremely uneven and relatively large dispersion. The coefficients of variation of Cu, Ni, Zn, Cd, and Pb were less than 50%, showing that it had a medium variability, comparatively uniform spatial distribution, and small fluctuations. The kurtosis coefficients of Sb, Zn, Cu, and Pb were between 2.3 and 3. The absolute values of the coefficient of skewness were between 0.0037 and 0.1552, which is consistent with the normal distribution. The absolute skewness coefficients of Ni and Cd were more than 0.32 and the coefficients of kurtosis were slightly lower, roughly belonging to the negative skewed distributions. These results demonstrated that the mining, smelting, waste slag stacking and transportation, human activities, and other factors possibly led to the difference in the metal content of the surface runoff in different regions.

### 3.3. Spatial Variation of Heavy Metals in Surface Runoff

#### 3.3.1. Correlation of Spatial Structure

**Figure 3:**
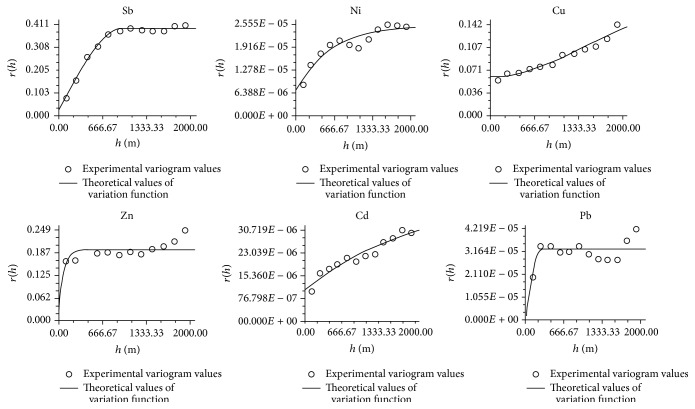
Theoretical semivariation function of Sb, Ni, Cu, Zn, Cd, and Pb in surface runoff in the antimony mine.

With an effective lag distance of 2 km and the tolerance angle of 22.5°, the variation functions of those Sb, Zn, Cu, Pb, and Cd resulting from the surface runoff of antimony mine area were calculated using GS+V9.0 software, respectively. The fitting of the theoretical model of semivariogram was conducted in hope of obtaining the best model as well as parameters (see Table 3). The fitting curve of the theoretical semivariation function is shown in Figure 3. As seen from Table 3 and Figure 3, the function fittings for the six kinds of the metals presented better performance, among which the fittings for the Sb, Zn, Ni, and Pb were better by the spherical model, while fittings for Cu, and Cd were better by the Gauss model. The coefficients of determination were between 0.611 and 0.974. The rate of nugget value to base sill value (*C*_0_/(*C*_0_ + *C*)) was 0.0767, closed to zero, indicating that the Sb in the surface runoff in the investigated area exhibited a strong spatial correlation and its spatial variability was mainly affected by the impact of regional factors, such as soil parent material and climate. *C*_0_/(*C*_0_ + *C*) of the Cd and Pb were 0.248 and 0.245, respectively, both of which were less than 0.25. This showed that the metals have a strong spatial correlation in a contaminated runoff in the investigated area, of which the spatial variability was dominantly affected by the regional factors. In addition, they were influenced by the random factors, such as industrial and agricultural production. *C*_0_/(*C*_0_ + *C*) values of the Ni, Cu, and Zn were 0.504, 0.3316, and 0.559, respectively, which indicated that Ni, Cu, and Zn had a moderate correlation degree, and the regional influence on spatial variability was the main factor; however, the impact of the random factor should not be ignored. The difference on the variable ranges of the six kinds of heavy metals was larger, wherein the shortest variable ranges for Zn were 178 m, which indicated that the spatial correlation within a small area was also strong. The variable range of Ni and Cd was 2001 m and 2579 m, respectively, which exceeded the scope of the investigated area. This implies that a significant drift phenomenon existed. Overall, the six kinds of metals showed an obvious “Nugget Effect.”

#### 3.3.2. Feature of Spatial Structure

**Figure 4:**
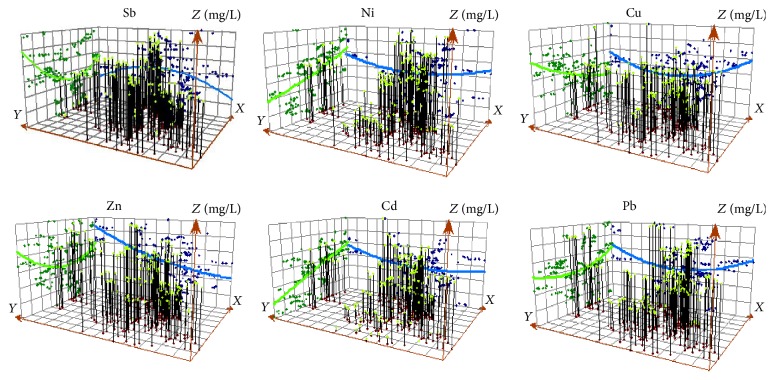
The spatial structures of Sb, Ni, Cu, Zn, Cd, and Pb in surface runoff in the antimony mine.

The spatial distributions of six kinds of the metals were analyzed in ArcGIS software platform; each of the vertical bars represented the concentration (height) and the position of one sample. These points were projected onto one orthogonal plane at east, west direction (*x*-axis) and north-south direction (*y*-axis). The optimal fitting line could be obtained through projecting points, through which the concentration at a particular direction could be simulated. The results are shown in Figure 4. As seen from the figure, except the Sb distribution roughly presenting an inverted “U” shape at north-south direction in blue line, other heavy metals are generally distributed in “U” shape. The Cd distribution showed the inverted “U” shape at east-west direction in green line, which was opposite to those of the other five kinds of metals. The Sb rapidly increased with north-south edge into the belly. In the investigated area at east-west direction, it showed that, on both sides of the direction, the concentration was higher than that at the middle. For those areas outside the investigated area, there would be a serious pollution by Sb. The distribution of Zn was similar to that of Sb at the east and west. The pollution increased along the south to north. There was no stable trend occurring at the end of the curve. This implies that the serious pollution occurred in the northern area. The concentration distribution of Cu on both sides of direction along north and south was higher than that of middle along north to south. A worsening pollution appeared along west to east. The distribution of Ni, Pb was similar to that of Cd, which had a uniform distribution with less variation at north and south, while the pollution increased along west to east.

#### 3.3.3. Directional Feature of Spatial Structure

**Figure 5:**
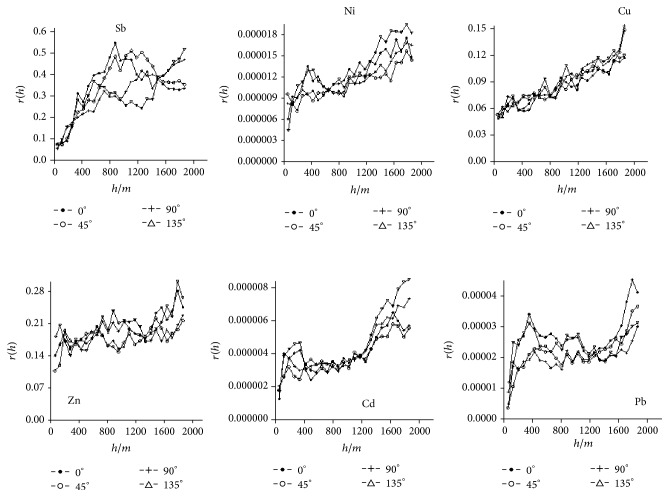
The direction of Sb, Ni, Cu, Zn, Cd, and Pb mutation function in surface runoff in the antimony mine.

The variability of the six kinds of metals have been calculated using the scientific software Suffer 8.0, and the figures at four directions (0° (E~W), 45° (NE~SW), 90° (S~N), and 135° (SE~NW)) and four kinds of broken lines were used to reflect the variations at different directions as shown in Figure 5. Figure 5 showed that the variations of Cu at four directions were approximately coincident, and the variations were almost the same in the step range from 0 m to 1900 m and an obvious anisotropy occurred. When the step was beyond 1900 m, the direction difference resulted in the different nugget values at each direction. The longest step range was 1907 m at 0° and the shortest step range was 1670 m at 90°, which all fell within the investigated area. The ratio of the nugget to sill between 0.2516 and 0.3382 showed a good spatial correlation. This also implied that Cu had a moderate variability within a larger variation range. Different models had different fitting effects for different directions, which indicated that the fitting effect by Gauss model was suitable for 0° and 90°, while that by the spherical and exponential models was suitable for 45° and 135°, respectively. The nugget, sill, and variable range values as well as the degree of variation at the direction (0°) are the highest. The fitting results showed that the variations of Zn, Ni, Pb, and Cd at the four directions were approximately coincident; besides, the variation of Ni was similar to that of Cd in the step range between 600 m and 1300 m, and the nugget value of Zn differed from that of Ni. Pb had a stronger variability within a wider variation range, which was different from Cu; Zn had a moderate variability within a smaller variation range; the correlation of Sb within a smaller variation range was very stronger. The distribution of Sb showed an obvious anisotropy in the step size of over 300 m, of which the difference was larger. The minimum variable ranges were at 0° and 90°, while the maximum variable range was 1276 m at 135°. The ratio value of the nugget to sill that was less than 0.25 could show a good spatial correlation. Gauss model could present a good fitting for 135°, while the spherical model was suitable for other three directions.

## 4. Spatial Distribution Characteristics

**Figure 6:**
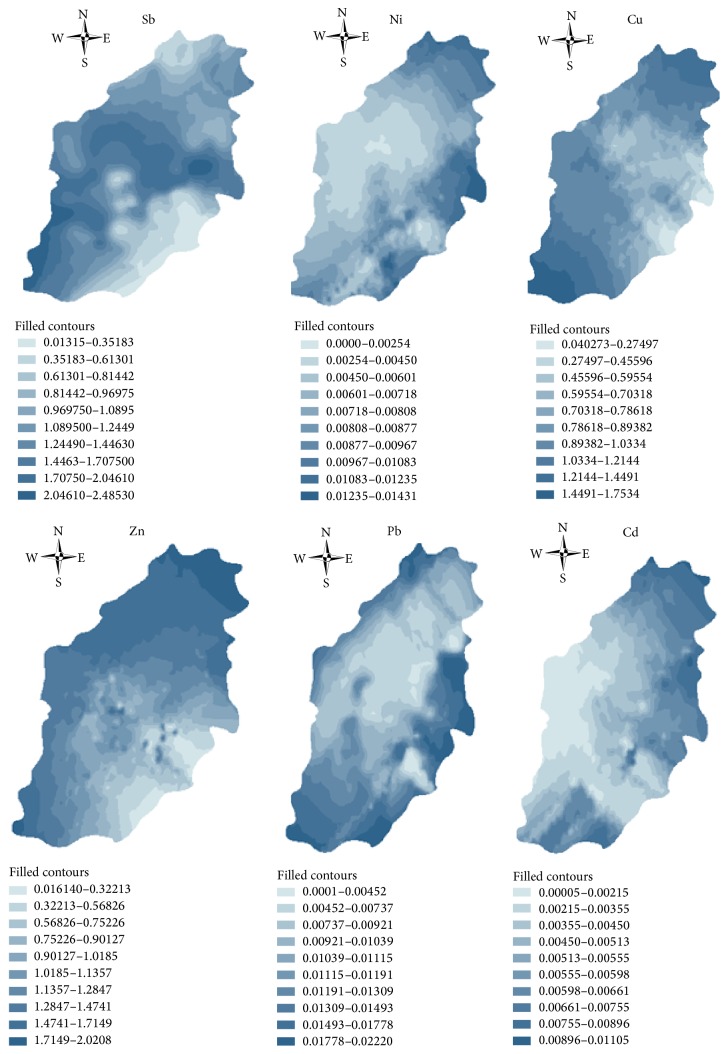
The predicted map of spatial distribution of Sb, Ni, Cu, Zn, Cd, and Pb in surface runoff in the antimony mine with Kriging interpolation.

Using ArcMap software, the distributions of the six kinds of metals were predicted by an interpolation method as the results shown in Figure 6.

First, from the figure, Sb was mainly concentrated in the southwest, central part, and northeast in the soil-water interface, forming three primary pollution source. The highest pollution values were in the central part. Combined with the sampling point distribution diagram, it could be seen that the widest area, southwest region, contains two mines, while the middle part and the northeast part contain one, respectively. The regions with the highest pollution levels roughly fell within those areas in the smelting plants. The concentration was gradually reduced from the middle part to north and south, while the Sb at both ends of the north and south was very low. In the northern zinc plant and southern Zhumu mountain with the Guangjia Ling as the boundary line, the soil-water interface on both ends of region was almost not polluted. This indicated that the pollution from Sb mainly came from the processes of mining and smelting. The smelting plant is located in the middle of the mine, which led to an enrichment of Sb in the smelting plant.

Second, the distributions of Cu and Zn were similar in the soil-water interface. The high concentration pollution was distributed on both sides of southern and northern areas. The low concentration pollution appeared in southeast, like a fan shape. Combined with sample points' distributions, two mines in the Quanqiu Village, a mine in Quanfengaojin and zinc plant in the north of Changlong boundary, form a sector arc shape. The Zn concentration inside the shape was obviously higher than that outside. Overall, the degree of pollution in the north was larger than that in the south. The regions with the highest concentration contained the zinc plant and station. The surrounding soil-water interface was polluted by waste gas and wastewater from factory through the hydrological cycle. The car tires, instrumentation, and organic compounds consisted of Zn and Cu. Thus, Cu and Zn in the pollution flow of the soil-water interference were expected to be primarily related to transportation, mining, and industrial activities in the investigated area. The pollution caused by those industrial activities and transportation to surrounding soil-water interface was much more serious than that by the mining activities.

Finally, the distributions of Ni, Pb, and Cd were similar in the interface of soil-water. These metals were mainly located in the northwest and southeast and distributed in the strip, while their concentrations tested in this study were very low in the southwest and northeast. The higher pollution levels were concentrated in Kuangshan country, Jiangda bay, and east of Huanggang Village. Their distributions were present in “U” shape at south-north direction and flat curve at west-east direction. Combined with the distributions of sampling points, the high pollution in the north fell within the residential district, station, and zinc plant; the high pollution in the east fell with the smelting plant; the high pollution in south fell with the residential district. The content of Ni was higher in alloy, electronic products, which mainly resulted from industry and infiltration and deposition of waste from mining. The Pb as an important material resulting from lead storage battery, alloy, pipe and cable, factory waste, and automobile exhaust presented a relatively higher content. The mining and smelting processes are also found to be an important source of Pb. Cd is an important material, which is produced by alloys, batteries, electronic products, and household appliances. There was a higher concentration in the waste of ore mining and industrial smelting. Therefore, the pollution of the three kinds of metals in the soil-water interface in the north would come from three aspects including the transportation, industry, and living activities. The pollution with high level in the east resulted from mining, smelting process and that in the south resulted from waste electronic products, household appliances, building materials, electroplating, and other types of living garbage.

## 5. Conclusions

In this study, the spatial variability and distributions of the metals in surface runoff in a nonferrous metal mine were investigated. The spatial distribution of the Sb in surface runoff in the investigated area was very uneven and relatively large dispersion. The coefficients of variation of Cu, Ni, Pb, Cd, and Cu were less than 50%, indicating a medium variability, uniform spatial distribution, and comparatively small fluctuations. Except the Ni concentration lower than the standard value, the maximum values of other five metals (Sb, Zn, Cu, Pb, and Cd) exceeded the standard values, which were 497.1, 2, 1.8, 22.2, and 22.1 times than the standard values, respectively. In addition, for all samples, the Sb concentrations were all over the standard values and for over half of the samples, Zn, Pb, and Cd exceed the standard values, while the Ni did not exceed the standard range. This showed that except Ni, the other five kinds of metals were the dominant pollution sources. The spatial structure characteristics of the six kinds of metals in soil-water interface pollution flow were analyzed. Sb, Zn, Ni, and Pb could be well fitted by the spherical model, and the Cd and Cu could be well fitted by Gauss model. The determination coefficients of the fitting model (*R* ^2^) were between 0.721 and 0.976, respectively, while the rate of nugget to sill value range from 0.0767 to 0.559 suggested that the six metals had a strong spatial correlation. The pollution distributions of Ni, Pb, and Cd were similar in the interfacial contamination of the soil-water, which was mainly concentrated in the ends of north and south and the east. The main reasons for contamination were attributed to residents living, transportation, and industrial activities. The distribution of Sb was mainly concentrated in the middle, resulting from smelting plant, mining, and industrial activities. The pollution distribution of Zn was similar to Cu, mainly concentrated on ends of the north and south and the west, of which the location fell within the mine, zinc plant, residential area, and traffic busy area. This indicated that the metals came from a wide range of sources.

## Figures and Tables

**Table 1:** The samples recovery rate of standard recovery experiment.

Heavy metal	Content (mg/L)			Recovery rate (%)
	Before	With scalar	After	
Sb	1.1031	0.5000	1.6021	99.8
Zn	0.9930	0.5000	1.4875	98.9
Cu	0.6598	1.0000	1.6718	101.2
Ni	0.0061	1.0000	1.0071	100.1
Pb	0.1867	1.5000	1.6792	99.5
Cd	0.0895	1.5000	1.5565	97.8

**Table 2:** Statistical characteristics of Sb, Ni, Cu, Zn, Cd, and Pb concentrations in surface runoff in antimony mine (mg/L).

Metal	Minimum	Maximum	Standard value	Median	Average	Standard deviation	Coefficient of variation	Geometric mean	Coefficient of skewness	Coefficient of kurtosis	Excessive standard rate	Distribution
Sb	0.1315	2.4853	0.005	1.1324	1.1599	0.5865	0.5056	0.7046	0.1552	2.3609	100%	Normal distribution
Ni	0.0001	0.0143	0.02	0.0078	0.0071	0.0034	0.4789	0.0049	−0.3208	2.3528	0	Negative skewness distribution
Cu	0.0403	1.7534	1	0.8193	0.7988	0.3255	0.4075	0.6791	−0.0982	2.8553	28.43%	Normal distribution
Zn	0.9161	2.0209	1	1.002	1.006	0.4619	0.4591	0.6315	0.0037	2.3398	50.51%	Normal distribution
Cd	0.0001	0.1105	0.005	0.0051	0.0047	0.0023	0.4894	0.0043	−0.3576	2.4736	53.43%	Negative skewness distribution
Pb	0.0001	0.2220	0.01	0.011	0.0111	0.0051	0.4595	0.0798	−0.0392	2.6395	60.29%	Normal distribution

**Table 3:** Theoretical semivariation function model and parameter of Sb, Ni, Cu, Zn, Cd, and Pb content of in surface runoff in the antimony mine.

Item	Type	*C*_0_	*C*_0_ + *C*	*C*_0_/(*C*_0_ + *C*)	Variable range (m)	*R* ^2^
Sb	Spherical	0.0290	0.3780	0.0767	918	0.956
Ni	Spherical	0.00000691	0.0000139	0.504	2001	0.782
Cu	Gauss	0.0601	0.1812	0.3316	1968	0.974
Zn	Spherical	0.0891	0.2022	0.5590	178	0.721
Cd	Gauss	0.00000284	0.00001147	0.248	2579	0.946
Pb	Spherical	0.0000167	0.0000681	0.245	1877	0.611
